# DEPDC5-Related Familial Focal Epilepsy With Variable Foci-1: A Report of a Rare Case

**DOI:** 10.7759/cureus.84627

**Published:** 2025-05-22

**Authors:** Asmita Itani, Prakash Banjade, Upama Shrestha, Salim Surani, Munish Sharma

**Affiliations:** 1 General Medicine, Institute of Medicine, Tribhuvan University, Kathmandu, NPL; 2 Pediatrics, One Brooklyn Health/Brookdale Hospital Medical Center, New York, USA; 3 Pediatrics and Child Health, Institute of Medicine, Tribhuvan University, Kathmandu, NPL; 4 Pediatrics and Child Health, Kanti Children's Hospital, Kathmandu, NPL; 5 Anesthesiology, Mayo Clinic, Rochester, USA; 6 Medicine, Texas A&M University, College Station, USA; 7 Medicine, University of North Texas, Dallas, USA; 8 Internal Medicine, Pulmonary Associates of Corpus Christi, Corpus Christi, USA; 9 Clinical Medicine, University of Houston, Houston, USA; 10 Pulmonary and Critical Care, Baylor Scott & White Medical Center - Temple, Temple, USA

**Keywords:** anti-seizure medications, depdc5 gene, electroencephalogram, family history, genetic epilepsy

## Abstract

Familial focal epilepsy with variable foci-1 (FFEVF1) is a genetic epilepsy syndrome associated with a pathogenic mutation in the DEPDC5 gene. It has autosomal dominant inheritance, along with incomplete penetrance and a variable phenotype. We present a case of focal seizures that progressed to generalized tonic-clonic seizures within the span of one year in a 12-month-old male child. A family history of epilepsy was present in the mother and grandmother of the child. Generalized epilepsy was observed in the initial EEG, while the MRI of the brain was unremarkable. Levetiracetam was unable to control the seizures; however, they were partially responsive to sodium valproate, which was prescribed later. A heterozygous pathogenic variant was revealed in exon 26 during whole-exome sequencing of the DEPDC5 gene. Family history and genetic testing can play crucial roles in pediatric epilepsy diagnosis, particularly when lab investigations and neuroimaging are normal, as showcased in this case.

## Introduction

The DEPDC5 gene (which encodes dishevelled, Egl-10, and Pleckstrin domain-containing protein 5) is located on chromosome 22q12.3 and spans approximately 154 kilobases. It encodes a protein composed of 1,603 amino acids [1]. DEPDC5 mutation-related epilepsy is characterized by overactivity of the mammalian target of rapamycin complex 1 (mTORC1) signaling pathway and is primarily focal epilepsy. Pathogenic germline mutations in the DEPDC5 gene, along with additional somatic mutations in brain tissue, are responsible for its causation. Familial focal epilepsy with variable foci-1 (FFEVF1) is a rare form of genetic epilepsy that is inherited in an autosomal dominant fashion. According to current research, approximately 13% of inherited epilepsy syndromes, including FFEVF, and 5% of nonlesional, sporadically occurring focal epilepsy consist of DEPDC5-related mutations [2,3].

This case report describes a young child with a positive family history of seizures, who presented with evolving seizures caused by a pathogenic variant of DEPDC5. The variant of the DEPDC5 gene mutation in this 12-month-old male child was extremely rare, and its population frequency was lacking in major databases. This report also highlights the significance of genetic testing in the diagnosis of epilepsy with unremarkable imaging and the implications of DEPDC5-related epilepsy in guiding therapeutic approaches and prognosis.

## Case presentation

A 12-month-old male child presented with frequent episodes of vacant stare, upward eye deviation, blinking of the left eye during the episodes, and clonic movement of the left side of the cheek, without loss of consciousness, indicating a seizure attack. The episodes lasted a few seconds and occurred every two to three days, with two to three episodes per day. The child was born to non-consanguineous parents and was the younger of two male siblings. The child’s mother and maternal grandmother also had a history of seizures, though neither has undergone genetic testing. The child's mother was on carbamazepine after the diagnosis of epilepsy at the age of 15 years. She had a seizure episode during her first pregnancy but was seizure-free during this pregnancy.

**Figure 1:**
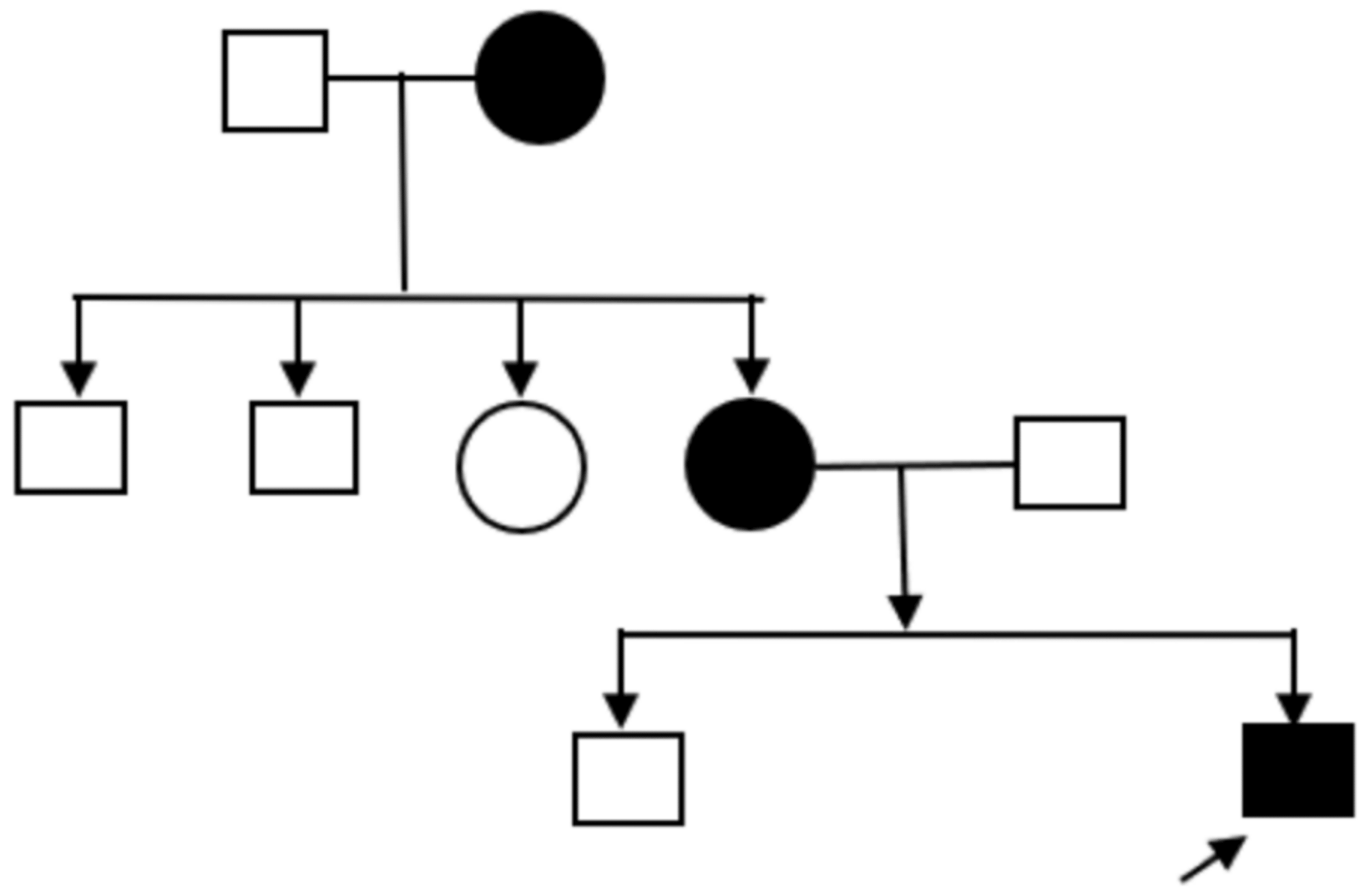
Family pedigree showing three generation inheritance of epilepsy ⬜ denotes unaffected male; ○ denotes unaffected female; ⬛ denotes affected male; ● denotes affected female; → denotes proband

The child was delivered at 37 weeks of gestation via lower-segment cesarean section (LSCS), due to a previous cesarean delivery. The pregnancy was otherwise uneventful. His birth weight was 2.5 kg. The neonatal period was uneventful, with no history of birth asphyxia, neonatal sepsis, jaundice, or NICU admission. Figure 1 depicts the pedigree chart of the family.

**Figure 2:**
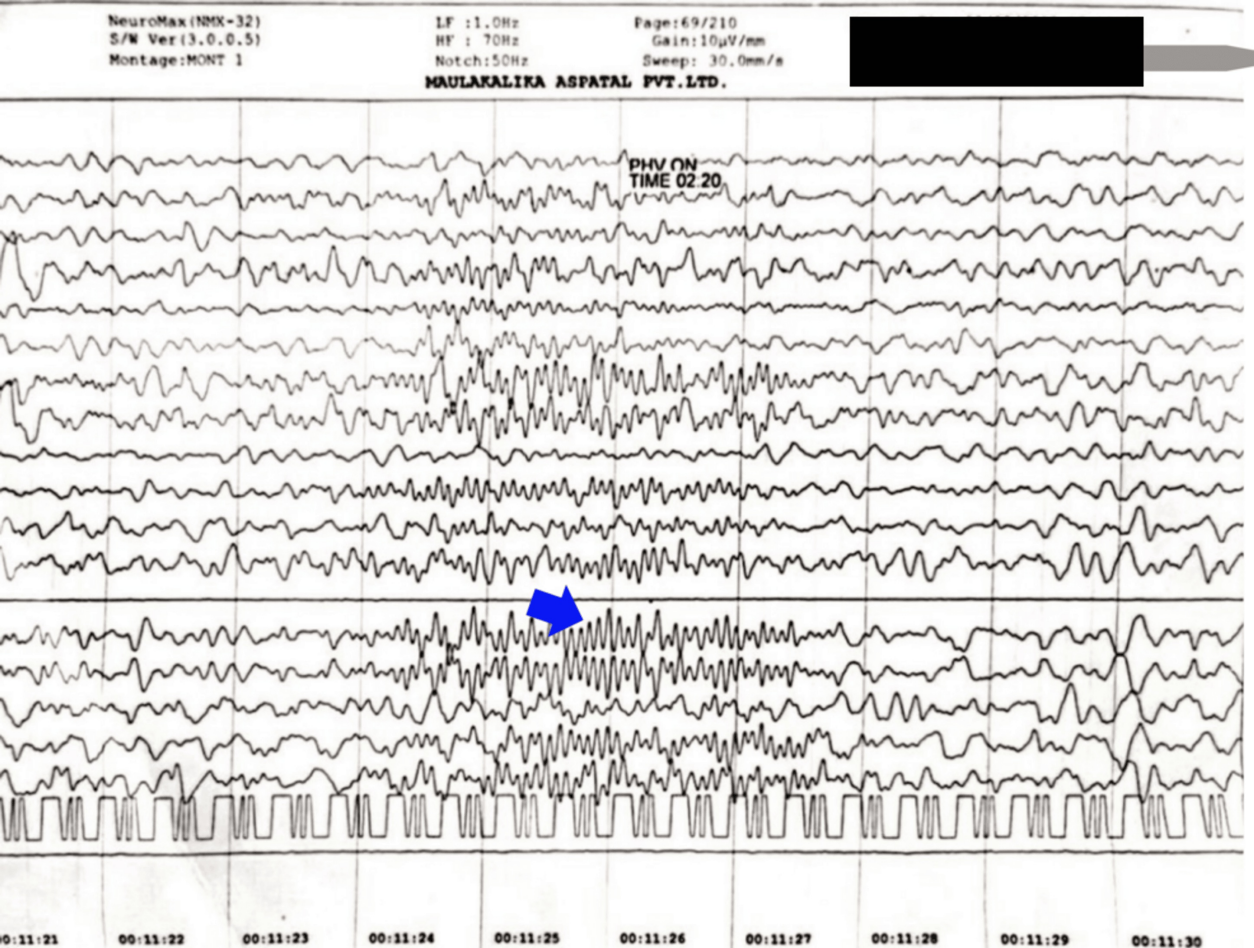
An electroencephalogram showing generalized epileptiform discharges (blue arrow)

The first EEG (30 minutes) was done at six months of age, on August 8, 2022, and showed generalized epileptiform discharges with sharp waves of 10-12 Hz spikes lasting two to three seconds, mainly arising from the left side, with occasional spread to the right (Figure 2).

The lab investigations, including complete blood count, electrolytes, glucose, renal function tests, liver function tests, ammonia, plasma lactate, and creatine kinase, yielded no diagnostic findings. The child was started on levetiracetam. The second EEG (30 minutes), done on December 8, 2022, was reported as normal. The child visited again on February 20, 2023, for ongoing seizures and sleep disturbances. The epilepsy protocol MRI of the brain, including T1-, T2-, and FLAIR-weighted sequences, showed no abnormality. Sleep EEG (30 minutes) on February 20, 2023, showed a generalized spike wave of 1-2 Hz and 100-200 microvolts. The child was started on sodium valproate, and midazolam nasal spray was prescribed for use in case of a seizure at home. Clobazam was also added to address sleep issues. A DNA test with whole-exome sequencing from blood samples detected pathogenic gene variants causing the reported phenotype. A heterozygous one-base pair duplication in exon 26 of the DEPDC5 gene on chromosome 22 was reported, resulting in a frameshift mutation and premature truncation of the protein 30 amino acids downstream of codon 733 (Table 1). The ClinVar database has classified the variant as pathogenic [4]. The child was then followed up monthly for the first six months and every three months for the next six months. During this one year of follow-up, the seizure episodes remained well-controlled, and sleep was significantly improved.

**Table 1:** Summary of DNA test for pathogenic gene variant FFEVF1: Familial Focal Epilepsy With Variable Foci 1

Gene (Transcript)	Location	Variant	Zygosity	Disease	Inheritance	Classification
DEPDC5 (+) (ENTST00000651528.2)	Exon 26	c.2196dup (p.Val733SerfsTer30)	Heterozygous	FFEVF1 (OMIM#604364)	Autosomal dominant	Pathogenic

## Discussion

DEPDC5 interacts with nitrogen permease regulator-like proteins (NPRL2 and NPRL3) and forms the GATOR1 complex (GAP activity toward Rags 1), which plays a crucial role in suppressing mTORC1. This complex regulates cell growth by detecting nutrient availability - particularly amino acids - through downstream effectors like P70S6K (p70 ribosomal protein S6 kinase 1) [5]. Recent studies have linked mutations in several genes involved in the mTOR signaling pathway - such as TSC1, TSC2, MTOR, DEPDC5, NPRL2, and NPRL3 - to the development of focal epilepsies in affected individuals [6]. Most mutations cause premature protein truncation, suggesting haploinsufficiency as the primary disease mechanism [7]. In a study involving 479 individuals diagnosed with focal epilepsy, eight unrelated participants were identified as carrying distinct pathogenic or likely pathogenic variants in the DEPDC5 gene. This corresponds to a prevalence rate of approximately 1.67% for DEPDC5-associated focal epilepsy within the study population [8].

DEPDC5-associated epilepsy has an autosomal dominant inheritance. Studies have shown incomplete penetrance and phenotypic heterogeneity associated with DEPDC5-related epilepsies. Therefore, genetic testing is essential, even without a known family history [8]. Interestingly, in one case, the affected child had unaffected parents [9]. This could have resulted from the low penetrance estimated by earlier family studies - between 45% and 67% [10,11]. De novo mutations and transmission from asymptomatic mosaic parents have also been observed. Although DEPDC5 mutations follow an autosomal dominant pattern, it is still unclear why many carriers remain unaffected [9]. Genetics, environment, and lifestyle are the probable factors influencing incomplete penetrance [12].

In our case, the presence of a known pathogenic DEPDC5 variant in the child and a history of epilepsy in three generations on the maternal side strongly suggest a familial epilepsy syndrome. Although genetic testing was unavailable for the mother and grandmother, the clinical pattern supports a diagnosis within the spectrum of FFEVF. This case highlights the importance of family history in recognizing genetic epilepsy syndromes, even when comprehensive genetic testing is not feasible. In our case, genetic testing helped confirm the etiology in the context of normal laboratory and neuroimaging results. This prevented unnecessary investigations, helped clinicians provide more targeted management, and enabled appropriate genetic counseling for the parents early in the course. The p.Val733SerfsTer30 variant has been classified as pathogenic by the ClinVar database [4]. This variant has not been reported in the 1000 Genomes, gnomAD (v3.1), gnomAD (v2.1), dbSNP, or TOPMed databases. The absence of population frequency for DEPDC5 c.2196dup (p.Val733SerfsTer30) in major population databases suggests that it is extremely rare.

While most individuals with DEPDC5-related epilepsy maintain normal cognitive function, there have been cases where intellectual disability, autism spectrum disorder, or other psychiatric conditions are present. Though DEPDC5 mutations are primarily associated with non-lesional epilepsy, they have been identified in about 20% of individuals with structural brain abnormalities [2]. The mean age of seizure onset in individuals with these mutations was 12.5 years. Interestingly, our patient had seizure onset at the age of six months - much younger than the mean age of seizure onset reported in previous studies. Temporal and frontal lobe epilepsies were the most prevalent forms [7]. As the mTOR pathway is involved in brain development, it is hypothesized that patients with DEPDC5 mutations may have minute lesions. Still, positive MRI results are seen in only a few patients. In a study involving four families with DEPDC5 mutations, MRI scans of all patients showed no apparent abnormalities. The likely reason for the underreporting of lesions in DEPDC5-related epilepsy is that the lesions are small - either at the cellular or tissue level - making them difficult to detect [13]. These findings are similar to our case, where the brain MRI did not reveal any abnormalities.

The treatment of DEPDC5-related epilepsies is based on their phenotype and genotype characteristics, with conventional anti-seizure medications (ASMs) being the initial approach. However, the effectiveness of these medications varies, as some individuals respond well to first-line ASMs, while others are resistant to treatment. Currently, to our knowledge, there is no evidence indicating that any specific ASM is more effective in controlling seizures. In our case, the child showed a poor response to levetiracetam but responded well to sodium valproate and clobazam. Refractory epilepsy is present in approximately half of the patients with DEPDC5 mutations. Following resective surgery for epilepsy, about 80% of refractory epilepsy cases become seizure-free or show significant improvement [14]. In studies, mTOR inhibitors like rapamycin have demonstrated a disease-modifying effect and provide long-term seizure reduction [15]. Currently, DEPDC5 agonists that selectively target mTORC1 are also being studied [16]. The ketogenic diet (KD) may be utilized for treating refractory mTOR-related epilepsy, according to a recent study that found KD could block mTOR signaling in rats' brains [17].

## Conclusions

The DEPDC5 gene mutation has been identified in both familial and sporadic cases of epilepsy. It is inherited in an autosomal dominant fashion and exhibits phenotypic variability and incomplete penetrance. Thus, genetic testing is recommended even when there is no known family history of epilepsy. This case report discusses a young male child with a pathogenic variant of DEPDC5, who has a positive family history of seizure disorders. It emphasizes the importance of considering family history and conducting genetic testing in pediatric epilepsy cases, especially when laboratory investigations and neuroimaging results are normal. Early genetic testing can avoid diagnostic delays, help guide personalized treatment, and provide family counseling about prognosis and inheritance patterns.
